# Cognitive predictors of cervical cancer screening’s stages of change among sample of Iranian women health volunteers: A path analysis

**DOI:** 10.1371/journal.pone.0193638

**Published:** 2018-03-20

**Authors:** Mohammad Reza Miri, Mitra Moodi, Gholam-Reza Sharif-Zadeh, Hakimeh Malaki Moghadam, Maryam Miri, Ensiyeh Norozi

**Affiliations:** Social Determinants of Health Research Center, Department of Public Health, Faculty of Health, Birjand University of Medical sciences, Birjand, Iran; School of Nursing, University of Alabama at Birmingham, UNITED STATES

## Abstract

**Introduction:**

The uptake of Pap smear among Iranian women is low, resulting in a high rate of casualties from cervical cancer in Iran. The present study used the Health Belief Model (HBM) and the Stages of Change theory as theoretical frameworks for understanding the predictors of the behaviour of Iranian Women Health Volunteers (WHVs) with respect to cervical cancer screening.

**Methods:**

Data from the 1,253 WHVs were analyzed using path analysis to assess the effects of cognitive factors (including knowledge, perceived susceptibility to cervical cancer, perceived severity of cervical cancer, Pap smear benefits, Pap smear barriers, and Pap smear self-efficacy) on the stages of change for Pap-smear behaviour.

**Results:**

The majority of the respondents (71.5%) reported that they had not taken previous Pap smear tests; only 3% had received a regular Pap test. The perceived benefits to cervical cancer screening, the perceived barriers to cervical cancer screening, and the perceived self-efficacy to perform cervical cancer screening emerged as the predictors of cervical cancer screening’s stages of change; perceived threat to cervical cancer, however, did not.

**Discussion:**

Uptake of regular cervical screening for Iranian WHVs was very low. Different interventions, such as media campaigns and educational interventions could provide an opportunity to improve women's knowledge on cervical cancer and Pap test benefits, address any misconceptions or fears about the procedure of the Pap test, and finally increase the cervical screening uptake by Iranian women.

## Introduction

Cervical cancer is the fourth most common cancer in women worldwide, with an estimated 530,000 new cases that were detected in 2012. About 84% of the new cases were detected in women living in less developed regions. Every year, more than 270,000 women die from cervical cancer; more than 85% of these deaths occur in low- and middle-income countries[1].

In developed countries, the incidence and the mortality rates of cervical cancer have dramatically declined because of proper access to high-quality cervical cancer screening programmes [1, 2]. In developing countries, where there is a limited access to effective screening programmes and low uptake of Pap smear or pelvic examination, the disease is often not identified until it reaches a further advanced state and symptoms develop, resulting in a higher rate of death from cervical cancer [1]. In Iran, Incidence rate of cervical cancer is considerably low; however, the mortality rate of cervical cancer is about 44% because of cervical cancer patients are often diagnosed in the advanced stages in Iran [3].

In Iran, cervical cancer screening programmes are free of charge and widely available in the public health sector. However, literature suggests that the uptake for cervical screening in Iranian women remains low, resulting in a high rate of death from cervical cancer in Iran [3, 4]. The reasons of Iranian women for not obtaining a Pap test included: perceived barriers to cervical cancer screening, perceived low benefits to cervical cancer screening and perceived low self-efficacy to perform cervical cancer screening [4, 5].

One of the most important groups of people who can have an important role in promoting Iranian women’s screening behaviours is the Women Health Volunteers. The ‘Women Health Volunteers’ (WHVs) are well-respected and interested women of the community who have enough spare time for training and for disseminating health messages within their communities [6]. Unfortunately, research using a theory-driven approach to study the status of cervical cancer screening behaviour and the related factors among this important group is limited.

The stage of change theory is one of the most important theoretical frameworks that have been shown to be useful in assessing an individual's readiness to act on a health behavior. This theory acknowledges that people differ in their readiness to adopt new behaviors. People adopting a new behavior go through a series of stages of readiness to change, including: pre-contemplation, contemplation, preparation, action, maintenance, and relapse [7]. In the other hand, Health Belief Model (HBM) is one of the most important theoretical frameworks that have been shown to be useful in understanding and explaining preventive behaviors [8, 9]. HBM is based on the assumption that an individual’s belief in a disease, such as perceived susceptibility and perceived severity; combined with an individual’s belief in the effectiveness of the recommended preventive behavior, serve as predictors of individuals’ behavior [10]. The present study, therefore, used the Health Belief Model (HBM) [10] and the Stage of Change Theory [7] as theoretical frameworks for understanding the predictors of Iranian WHV’s behaviours with regard to cervical cancer screening.

## Methods

### Participants

This study was conducted in the Southern Khorasan Province, which is an eastern Iranian province, from June to November 2016. All the procedures were approved by the Ethics Committee of Birjand University of Medical Sciences (Ir.bums. 2016.35). The study participants were a sample of WHVs recruited from 11 cities of the Southern Khorasan Province. The WHVs programme is an integral part of Iran’s primary healthcare plan. WHVs must have a basic level of literacy that is equivalent to the completion of primary school. Volunteers are recruited principally (though not exclusively) from among married women. Women volunteers are empowered by their participation and are enabled to act as a bridge between healthcare services and their own communities [6]. Multi-stage random sampling was used to select the participants. Eleven cities of Southern Khorasan province served as the stratum and the numbers of women selected from each city were proportional to the size of the WHV’s population in each stratum. Subjects were included if they were married and aged between 20 and 65 years. Women with a history of hysterectomy or gynaecological cancer were excluded from this study. Of 1,627 eligible subjects recruited in this study, 1,410 (86.66%) consented to participate. The most common reason for refusal included the participant’s lack of willingness to be part of the research and his/her lack of interest in the study. The study was described to participants and they were assured that their participation was voluntary. Written informed consent was provided by all the participants.

### Measures

All the measures were self-administered, which required around 15-minute completion times. The following points provide a description of each measure:

1. The measurement of the socio demographic characteristics consisted of age (in years), age during first pregnancy, number of children, marital status, educational level, employment status, insurance type, and qualitative assessment of income.
2. The cervical cancer screening stages were measured by the single item that assessed the stages of change for cervical cancer screening behaviour; this item was adapted from the work of Tung et al. (2008) [2]. Each participant’s response was classified into one of the six stages: (a) pre-contemplation (never had a Pap test and is not thinking about having one in the next six months), (b) contemplation (never had a Pap test, but is thinking about having one in the next six months), (c) preparation (never had a Pap test, but is thinking about having one in the next month), (d) action (had one Pap test in the past and intends to continue), (e) maintenance (had regular Pap tests before and intends to continue), (f), and relapse (had Pap tests previously but has no intention to continue). Content validity of this single item was assessed by a panel of experts (four health educators, four gynecologists, and two society health nurses). Clarity of this item was also assessed by 20 women who were similar to the target group of this study.
3. The participant’s beliefs about cervical cancer and the Pap test were measured by a 23-item questionnaire that was based on the Health Belief Model (HBM) constructs; these included five sub scales: perceived susceptibility and perceived severity to cervical cancer, Pap smear benefits, Pap smear barriers, and Pap smear self-efficacy. The items in the perceived susceptibility subscale referred to a woman’s perceptions about her chances of getting cervical cancer on the basis of three items (for example: ‘My chances of getting cervical cancer in the next few years are great’). Perceived severity was measured by 6 items that assessed the female’s beliefs with reference to the severity of cervical cancer (for example: ‘Cervical cancer would threaten my relationship with my husband’). The category of Pap smear benefits was measured by a 4-item questionnaire that assessed women’s perceived benefits of obtaining a Pap test (for example: ‘If I go through a Pap test and nothing is found, I don’t need to worry much about cervical cancer’). The category of Pap smear barriers were measured by a 6-item questionnaire that assessed women’s perceived barriers towards obtaining a Pap test (for example: ‘Having a Pap test is too embarrassing’). Self-efficacy was measured by a 4-item questionnaire that assessed women’s confidence in getting a Pap test (for example: ‘I am confident that I can get a Pap test done even if it might be embarrassing). For each item, the respondents were asked to rate their beliefs on a 5-point Likert scale (ranging from 1 = Strongly Disagree to 5 = Strongly Agree). These items were adapted from the Champion Health Belief Model Scale (CHBMS) [11]. This questionnaire was examined in terms of features such as content validity and internal consistency. The content validity of the questionnaire (the Content Validity Ratio (CVR) and the Content Validity Index (CVI)) was determined by a panel of experts (four health educators, four gynecologists, and two society health nurses). The reliability of the each subscale of the questionnaire was calculated separately using Cronbach's alpha. A test of internal consistency indicated that Cronbach's alpha for the overall scale and each subscale of the questionnaire was adequate (α ≥ 0.70).
4. In addition, in order to assess an individual’s knowledge about Pap test, we used 4 items (participant’s knowledge of the recommended frequency of the Pap test, the best time to perform a Pap test, the start time of a Pap test, and the usage of a Pap test). Each correct response was scored one point and each wrong response was scored zero. Content validity of these items was assessed by a panel of experts (four health educators, four gynecologists, and two society health nurses). These items were piloted on 20 women in order to check the clarity of each item.

### Data analysis

The data were analyzed using the SPSS version 18.0 (SPSS Inc., Chicago, IL) and the AMOS version 22.0 software. The summary statistics (means, standard errors, frequencies, and percents) were used to describe the participant characteristics and the stages of change for Pap smear behaviour. The Pearson correlation coefficient was used to determine the linear relationship between each of the HBM constructs and the Pap smear behaviour score. Path analysis was used to identify the effects of the predictor variables on the stages of change in the Pap smear behaviour. Path model fitting was conducted with asymptotically distribution-Free methods. The model fit indices were examined to test the overall fit of the model to the collected data. The overall model goodness of fit was assessed through the chi-squared test, supplemented by comparative fit indices (Normed Fit Index [NFI] and Comparative Fit Index [CFI]) and an absolute fit index (Goodness-of-Fit Index [GFI]; in this index, values of 0.9 or over reflect a good fit) [12, 13]. In addition, the Root Mean Square of Approximation (RMSEA) was considered, in which values below 0.08 are considered acceptable. The estimated direct, indirect, and total effects were described in terms of standardized regression coefficients and they were evaluated using both of the Sobel tests [14].

## Results

Of the 1410 distributed questionnaires, only 1253 of the questionnaires were completely filled out. The incomplete questionnaires were excluded from the analysis. The respondents’ mean age was 35.67 ± 8.89 years; the mean age at the beginning of pregnancy was 21.88 ± 3.72 years; the mean age of marriage was 20.34 ± 3.72 years; and the number of children was 2.59 ± 1.28. A total of 93.5% of the respondents were married and 87.4% were housekeepers. Only 30.8% had high school diplomas and 13.5% had attended college. Most of the women (99.3%) had health insurance. Nearly three percent of the women (2/7%) reported a history of cervix cancer in their first-degree relatives. The majority of participants (68%) evaluated their economic situation as moderate. Most of respondents reported that they had heard of Pap smear testing (70%). Table 1 shows demographic characteristics of studied women in detail.

**Table 1:** Demographic characteristics of the participants (n = 1253).

Variable		Number (percent)
Age	20–30 y	379(30.2)
	30–40 y	515(41.1)
	40> y	359(28.7)
Marital status	Married	1171(93.5)
	Divorced or Separated	82(6.5)
Educational level	< High School Diploma	698(55.7)
	High School Diploma	386(30.8)
	Attended college	169(13.5)
Occupation status	Housekeeper	1070(87.4)
	Employed part-time	47(3.7)
	Employed full- time	110(9)
Economic situation	Poor	283(22.6)
	Moderate	852(68)
	Good	115(9.4)
Health insurance	Yes	1244(99.3)
	No	9(0.7)
History of cervix cancer in first-degree relatives	Yes	29(2.3)
	No	1224(97.7)
History of education about cervical cancer	Yes	27(2.1)
	No	1226(97.9)

The majority of the participants were classified into the preparation stage, followed by the action stage, the pre-contemplation stage, the contemplation stage, and the maintenance stage. There were no participants classified into the relapse stage (Table 2).

**Table 2:** Frequency distribution of Pap smear behavior among women health volunteer in Southern Khorasan province.

Stages of change	N (%)
Pre contemplation	279 (22.3)
Contemplation	262 (20.9)
Preparation	354 (28.3)
Action	320 (25.5)
Maintenance	38 (3)
Total	1253 (100)

Table 3 shows the means of the HBM constructs as well as the relationship between the model’s constructs and the Pap smear behaviour. According to the table, the relationships between the constructs were weak to moderate.

**Table 3:** Correlation matrix, means, and standard deviation of model constructs and Pap smear behavior (stage of change).

	M ± sd	Pap smear behavior	Self-efficacy	benefits	benefits	Threats	Knowledge
Pap smear behavior	2.66±1.17	1					
Perceived Self-efficacy	15.31±3.01	0.25**	1				
Perceived benefits	16.50±2.41	0.26**	0.42**	1			
Perceived barriers	49.4±13.98	-0.28**	-0.37**	-0.20**	1		
Perceived Threats	31.36±5.28	0.06*	0.14**	0.30**	0.02	1	
Knowledge	2.72±0.91	0.13**	0.09**	0.23**	-0.15**	0.019	1

**. Correlation is significant at the 0.01 level (2-tailed).

*. Correlation is significant at the 0.05 level (2-tailed).

**Fig 1:**
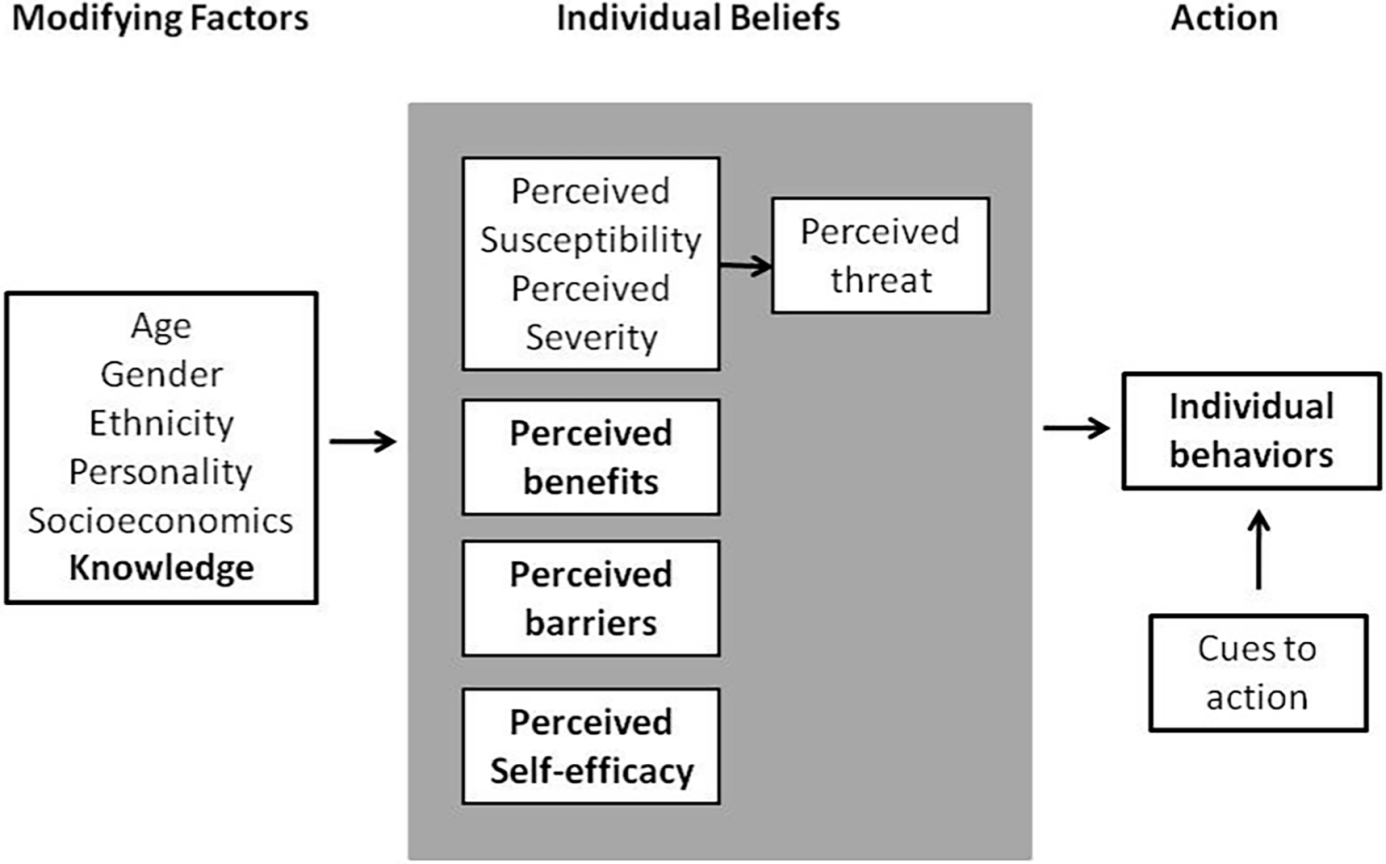
Health belief model components and linkages.

**Fig 2:**
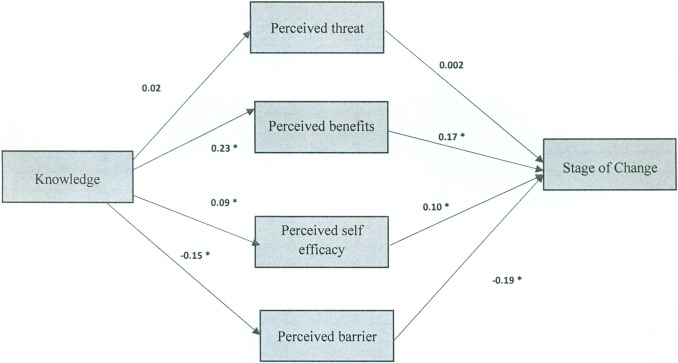
Path analysis model of cognitive predictors of Pap smear behavior (stage of change, R-squared = 0.10). *. Correlation is significant at the 0.01 level (2-tailed).

Path analysis was used to determine the effective constructs on Pap smear behaviour. The assumed model was designed based on the HBM conceptual model (Fig 1) [15]. The findings demonstrated that the final model had a reasonable fit with the data (Fig 2 and Table 4); this is shown as follows:

Χ2 (3) = 4.84 (P = 0.18), CMIN/DF = 1.61, GFI = 0.99, AGFI = 0.97, NFI = 0.99, CFI = 0.99, RMSEA = 0.05.

**Table 4:** Direct, indirect, and total standardized effects of HBM constructs on Pap smear behavior among women of Southern Khorasan province.

	Direct effect		Indirect effect		Total standard effect	
	β	P	β	P	β	p
Perceived benefits	0.17	0.01	-	-	0.17	0.01
Perceived barriers	-0.19	0.01	-	-	-0.19	0.01
Perceived Self-efficacy	0.10	0.01	-	-	0.10	0.01
Perceived Threat	0.002	0.99	-	-	0.002	0.99
Knowledge	-	-	0.08	0.01	0.08	0.01

As shown in Fig 2 and Table 4, the perceived benefits, the perceived barriers, and the perceived self-efficacy have direct and significant effects on Pap-smear behaviour. The perceived threat has no direct effect on Pap-smear behaviour. Given the beta coefficient for the total effect, the perceived barriers construct has the greatest impact on Pap smear behaviour.

## Discussion

Iran has a population of 30.13 million women aged 15 years and older, who are at risk of developing cervical cancer [16]. In Iran, where there is a high access to effective screening programmes, the uptake of Pap smear or pelvic examination is low, thereby resulting in a high rate of death from cervical cancer [3, 4, 16]. Therefore, there is a pressing need to increase the uptake of cervical cancer screening among Iranian women. An understanding of factors that predict cervical cancer screening behaviour may contribute to the development of more effective screening programmes. The present study used the Health Belief Model (HBM) and the Stages of Change theory as theoretical frameworks for understanding the predictors of the behaviour of Iranian WHVs with regard to cervical cancer screening.

As seen in the prior ‘Results’ section, the majority (71.5%) of the respondents reported not having gone through previous Pap smear testing; only 3% reported receiving a regular Pap smear test. The regular use of the Pap test was also low in other developing countries [17, 18]. For example, only 7% of the nursing staff in a tertiary-level teaching institution in rural India had been screened by the Pap test [18]. In developed countries, where there is proper access to effective cervical cancer screening programmes, the regular use of the Pap test is higher than in developing countries [2, 19], resulting in a lower rate of death from cervical cancer in these countries [1]. In Iran, cervical cancer screening programmes are free of charge and are widely available in the public health sector. However, several potential factors may explain the low uptake of regular Pap test among our study group. In this study, we examined the role of theory-based cognitive factors in the uptake of the regular Pap test. According to our results, the perceived benefits, the perceived barriers, and perceived self-efficacy emerged as predictors of cervical cancer screening’s stages of change among Iranian WHVs. According to the stages of Pap test adaptation, women in later stages (for example: maintenance) demonstrated higher levels of perceived benefits, higher levels of perceived self-efficacy, and lower levels of perceived barriers than women in the earlier stages (for example: pre-contemplation).

Although a perceived benefit was predictor of the stages of change for the Pap test, unfortunately the results of our study showed that most of the participants had no information about the approaches to cervical cancer prevention and their benefits. Indeed, only 2.1% of the respondents of our study stated that they have been trained on the importance of the Pap test for the early detection of cervical cancer. In addition, about 30% of the respondents stated that they had never heard of the Pap smear test. These issues highlight the weaknesses of the Iran health centers in terms of their failure to disseminate information on the disease and training issues associated with cervical cancer. The finding which established that the perceived benefits significantly predicted the stages of change for the Pap test is in line with our own hypothesis as well as the findings from several prior studies [2, 5, 18, 20–23]. For example, Jia et al. (2013) showed that the women who obtained information about cervical cancer and the Pap test were more sensitive to it. They also have greater motivation, higher benefit perception, and less barrier perception[23]. This suggests the need for continued efforts to increase one’s awareness of the benefits of the Pap test among Iranian women. In this regard, holding well-planned educational classes about the importance of the Pap test, the method to conduct the test, and its effectiveness is suggested. On the other hand, the Iran mass media attention towards introducing effective screening programmes is low. In this regard, campaigns for promoting cervical cancer screening uptake could be channeled through mass media. These campaigns, in addition to improving women's knowledge about cervical cancer as well as Pap test benefits, can be an excellent opportunity to address the misconceptions or fears about the procedure of the Pap test, and finally encourage women to received Pap smears.

Consistent with previous TTM research on the use of the Pap smear [2, 5, 20, 24], perceived barriers and perceived self-efficacy were also among the determinants of progress in the stages of Pap test adaptation. The perceived barriers are those tangible and psychological costs associated with the Pap test that may prevent individuals from taking preventive action [25]. Several barriers to cervical cancer screening have been reported by women from different countries; some of them included: fear of pain [26], fear of positive Pap test results [4], having no health insurance [8, 27, 28], financial constraint [4, 28, 29], embarrassment about undergoing a Pap smear test [24], lack of time [4, 24], lack of knowledge about cervical cancer and the need for cervical screening [4, 18, 23, 24, 26, 29, 30] and fatalistic attitudes about cervical cancer and other aspects of health [23, 26, 29]. Based on results of our study the most important perceived barriers to cancer screening included: embarrassment about undergoing a Pap smear test, lack of time for undergoing a Pap smear test, fear of Pap test pain, fear of positive Pap test results, doubts about Pap test effectiveness, and fear about the procedure of a Pap smear test. These possible barriers can diminish women’s self-efficacy of being able to participate in future screening, so must be reduced as much as possible. Improving patient–provider communication can be an excellent approach to address the misconceptions or fears about the procedure of the Pap test. In addition, holding well-planned group discussions about the misconceptions or the fears about the procedure of the Pap test have been suggested.

## Conclusion

Unfortunately, the uptake of regular cervical screening for Iranian women health volunteers was very low. Several behavioural change theories like the Health Belief Model (Becker, 1974) and the Theory of Planned Behaviour (Ajzen, 1991) highlight the importance of individual perception in achieving behavioural change [25, 31]. Unfortunately, Iranian women have high barrier perception toward uptake of cervical screening. As mentioned above, in Iran cervical cancer screening programmes are free of charge and widely available in the public health sector. Therefore, the cost of services and access to services are not barriers for women to use this service. In fact, the barriers of Iranian women for not having regular Pop test are often psychological barriers such as: embarrassment about undergoing a Pap smear test, lack of time for undergoing a Pap smear test, fear of Pap test pain, fear of positive Pap test results, doubts about Pap test effectiveness, and fear about the procedure of a Pap smear test. Most of these barriers are cognitive and can be addressed by effective training. Different interventions, such as media campaigns, educational interventions, using mobile units, group discussions, etc. will provide an opportunity to improve women's knowledge about cervical cancer and the Pap test benefits in order to address the misconceptions or their fears about the procedure of the Pap test, and finally, to increase the cervical screening uptake by Iranian WHVs.

## Limitations

Several limitations in this study should be acknowledged. The first limitation relates to the fact that the several possible factors that motivate women to uptake the Pap test were not addressed in our study. Indeed, human behaviour is increasingly recognized as a complex product of the dynamic interactions between many more than the few factors that were considered in this study. For example, the results of one critical review proposed a variety of factors other than those specified by the HBM, which were successful predictors of women’s screening behaviour; these included their previous healthcare experiences, the social influence of their family members and physicians, a sense of fatalism, and contextual factors such as screening as part of a general health check-up [32]. Other individualistic and social/environmental variables of interest should be considered in future studies.

On the other hand, the sample of this study was composed solely of female health volunteers of the Southern Khorasan Province. The results of this study should not, therefore, be generalized to the WHVs of the other provinces of Iran. Further research should be conducted on the other samples of the WHVs from different socio cultural backgrounds. As the design of this study was cross-sectional, it is difficult to establish any causal association between women's beliefs of cervical cancer risk and their future regular uptake of the Pap smear test. In addition, the possibility of over-report or under-report by the samples should be taken into account.

## Supporting information

S1 File HBM Questionnaire in English language. (DOC)

S2 File HBM questionnaire in original language. (DOC)

S3 File Data File. SPSS (18.0).
(SAV)
