# Supplementary material for: Cognitive predictors of cervical cancer screening’s stages of change among sample of Iranian women health volunteers: A path analysis
Source: PLoS One. 2018 Mar 20;13(3):e0193638. doi: 10.1371/journal.pone.0193638 (PMC5860704; doi:10.1371/journal.pone.0193638)
Supplement: S1 File — (DOC) [file pone.0193638.s001.doc]

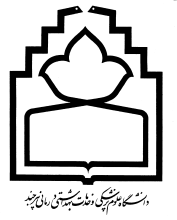

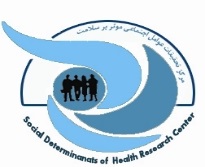
**Birjand university of Medical Sciences Social Determinants of Health Research Center**

*Dear Respondent,*

*The present questionnaire is part of a research project on* ***"Comprehensive Women's Cancer Screening Program"****. The questionnaire is anonymous and its information will be kept confidential. The results of this research will only be used for scientific purposes and will not have any negative consequences for you.*

*Many thanks in advance for your cooperation,*

***Birjand University of Medical Sciences***

**1-** **Date of birth:** ....................................

**2- Education** **Level:** Elementary School □ Middle School □ Secondary School □ High School Diploma □

Associate Degree □ Bachelor’s Degree □ Master's Degree and Higher □

**3-** **Marital Status:** Married □   Divorced □  Widowed □

**4-** **Occupation:**  Housewife □  Working at Home □     Working Outside □

**5-** **Age at Marriage:** ...............        **5.1** **-** **Age at First Pregnancy:** ..............        **5.2 - Number of Children:** ..................

**6-** **How do you rate your income status?** Good □  Average □  Poor □

**7-** **Health Insurance Coverage:**  Insured □  Uninsured □

**8-** **Does anyone in your first-degree relatives (mother, sister, daughter) have a history of cervical cancer?** Yes No

**9-** **Do you have any information about cervical cancer and its diagnostic methods?**  Yes□  No□

**10-** **If yes, identify the source of your information in priority order by numbers.**

Health workers□  Physician□  Radio/TV□  Book□  Magazines□  People with cancer□

Friends and relatives□  Internet□  In-person training□  Responsive SOS call□ Other sources□ .........................

**11- What** **do you think the Pap smear test is used for?**

Diagnosis of cancer □      Diagnosis of infection □     Both cases □     I have no idea □

**12**- W**hen should the Pap smear test be done for the first time**?

Since the first menstruation □        Since marriage □        Upon reaching menopause □        It can be done at any age □

**13- What is the** **best time to perform the Pap smear test?**

    Early days of menstruation□ The last day of menstruation □ 4-5 days after the end of menstruation□

2 weeks after the end of menstruation □

**14-** **After performing the Pap smear test, how often should it be repeated?**

     Every one year □  Every two years □ Every three years □

**Please select an option for each of the following statements.**

| **Perceived Sensitivity** |  | **Strongly Disagree** | **Disagree** | **No idea** | **Agree** | **Strongly Agree** |
| --- | --- | --- | --- | --- | --- | --- |
|  | | | | | | |
| 1. There is a risk of cervical cancer for all women. | |  |  |  |  |  |
| 2. The risk of cervical cancer is high in me in the next few years. | |  |  |  |  |  |
| 3. There is a risk of cervical cancer at any age. | |  |  |  |  |  |

| **Perceived Severity** |  | **Strongly Disagree** | **Disagree** | **No idea** | **Agree** | **Strongly Agree** |
| --- | --- | --- | --- | --- | --- | --- |
| 1. Cervical cancer is a dangerous disease. | |  |  |  |  |  |
| 2. Cervical cancer is fatal. | |  |  |  |  |  |
| 3. If someone suffers from cervical cancer, her sexual relationship will be affected. | |  |  |  |  |  |
| 4. Even thinking about suffering from cervical cancer scares me. | |  |  |  |  |  |
| 5. Cervical cancer leads to disease-related problems for a long time. | |  |  |  |  |  |
| 6. Cervical cancer disrupts the person's life. | |  |  |  |  |  |

| **Perceived Benefits of Pap Smear Test** |  | **Strongly Disagree** | **Disagree** | **No idea** | **Agree** | **Strongly Agree** |
| --- | --- | --- | --- | --- | --- | --- |
| 1. Pap smear test contributes to early detection of cervical cancer. | |  |  |  |  |  |
| 2. Pap smear test reduces the risk of death from cervical cancer. | |  |  |  |  |  |
| 3. Performing a Pap smear test will relieve my stress. | |  |  |  |  |  |
| 4. Pap smear test can lead to early detection of uterine infections. | |  |  |  |  |  |

| **Perceived Barriers of Pap Smear Test** |  | **Strongly Disagree** | **Disagree** | **No idea** | **Agree** | **Strongly Agree** |
| --- | --- | --- | --- | --- | --- | --- |
| 1. I’m embarrassed to get a Pap smear test. | |  |  |  |  |  |
| 2. I do not have enough time to go for Pap smear test. | |  |  |  |  |  |
| 3. Pap smear test is painful for me. | |  |  |  |  |  |
| 4. I'm afraid of performing a Pap smear test because I do not know what it will be like. | |  |  |  |  |  |
| 5. I do not go for the Pap smear test because I fear the result might be positive. | |  |  |  |  |  |
| 6. I'm skeptical about the effectiveness of pap smear for preventing from cervical cancer. | |  |  |  |  |  |

| **Perceived Self-Efficacy for Pap Smear Test** |  | **Strongly Disagree** | **Disagree** | **No idea** | **Agree** | **Strongly Agree** |
| --- | --- | --- | --- | --- | --- | --- |
| 1. I can easily perform a Pap smear test. | |  |  |  |  |  |
| 2. I can easily adjust my time to perform a Pap smear test. | |  |  |  |  |  |
| 3. I can easily overcome my fear of performing a Pap smear test. | |  |  |  |  |  |
| 4. I can easily overcome my embarrassment of performing a Pap smear test. | |  |  |  |  |  |
