# Supplementary material for: Cognitive predictors of cervical cancer screening’s stages of change among sample of Iranian women health volunteers: A path analysis
Source: PLoS One. 2018 Mar 20;13(3):e0193638. doi: 10.1371/journal.pone.0193638 (PMC5860704; doi:10.1371/journal.pone.0193638)
Supplement: S2 File — (DOC) [file pone.0193638.s002.doc]

**
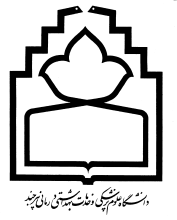
**


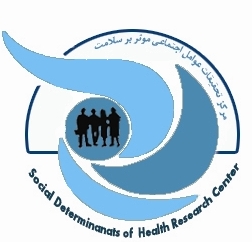


**به نام خدا**

با سلام و احترام، دوست عزيز پرسشنامه اي كه در دست داريد مربوط به طرح تحقيقاتي در زمينه **" طرح جامع غربالگری سرطان های شایع زنان "** است. اين پرسشنامه بدون نام بوده و اطلاعات آن نزد مجري طرح محفوظ خواهد ماند. نتايج اين تحقيق فقط به منظور اهداف علمي استفاده خواهد شد و براي شما هيچ گونه تبعات منفي نخواهد داشت. پيشاپيش از همكاري شما سپاسگزاريم. **با تشكر- دانشگاه علوم پزشکی بیرجند**

**1- تاریخ تولد:** ....................................

**2- میزان تحصیلات:** ابتدایی□ راهنمایی□ متوسطه□ دیپلم□ فوق دیپلم□ لیسانس□ فوق لیسانس و بالاتر□

**3- وضعیت تاهل:** متاهل□ مطلقه□ همسر از دست داده□

**4- شغل:** خانه دار□ شاغل در منزل□ شاغل در بیرون از منزل□

**5- سن ازدواج:** ................. **6- سن اولین حاملگی:** .......................  **7- تعداد فرزندان:** ..........................

**6- وضعیت درآمد خود را چگونه ارزیابی می کنید؟** خوب□ متوسط□ ضعیف□

**7- وضعیت بیمه درمانی:** بیمه هستم□ بیمه نیستم□

**8- آیا در بستگان درجه یک شما (مادر، خواهر، دختر ) سابقه ابتلا به سرطان دهانۀ رحم وجود داشته و یا دارد**؟ بلی□ خیر□

**9- آیا اطلاعاتی در رابطه با سرطان دهانۀ رحم و راههای تشخیص به موقع آن دارید؟** بلی□ خیر□

**10- در صورت مثبت بودن منبع کسب اطلاعات خود را به ترتیب اولویت با عدد مشخص کنید**؟

کارکنان بهداشتی□ پزشک□ رادیو تلویزیون□ کتاب□ مجلات□ افراد مبتلا به سرطان□ دوستان و آشنایان□ اینترنت□ آموزش حضوری□ تلفن امدادی پاسخگو□ سایر منابع□ ..............................

**11- به نظر شما آزمایش پاپ اسمیر به چه منظور به کار میرود؟** تشخیص سرطان□ تشخیص عفونت □ هر دو مورد □ نمی دانم□

**12- زمان شروع انجام ازمایش پاپ اسمیر کدامیک از موارد زیر است**؟

از شروع اولین قاعدگی □ از زمان ازدواج □ پس از رسیدن به سن یائسگی □ در هر سنی می شود مراجعه کرد □

**13- بهترین زمان برای انجام پاپ اسمیر کدام است؟**

اوایل خونریزی قاعدگی □ روز اخر خونریزی قاعدگی□ 4-5 روز پس از پاک شدن □ 2 هفته پس از پاک شدن□

**14- در صورت انجام پاپ اسمیر، هر چند وقت یکبار باید ان را تکرار کرد**؟ هر 1 سال □ هر دو سال یکبار□ هر سه سال یکبار□

**لطفا نظرات خود را در رابطه با عبارات زیر بیان بفرمایید.**

|  | **کاملاً مخالفم** | **مخالفم** | **نظری ندارم** | **موافقم** | **کاملاً موافقم** |
| --- | --- | --- | --- | --- | --- |
| **حساسیت درک شده** | | | | | |
| 1- احتمال ابتلا به سرطان دهانه رحم، برای همه زنان وجود دارد. |  |  |  |  |  |
| 2- احتمال ابتلای من به سرطان دهانه رحم در چند سال آینده زیاد است. |  |  |  |  |  |
| 3- احتمال ابتلا به سرطان دهانه رحم در هر سنی، وجود دارد. |  |  |  |  |  |

|  | **کاملاً مخالفم** | **مخالفم** | **نظری ندارم** | **موافقم** | **کاملاً موافقم** |
| --- | --- | --- | --- | --- | --- |
| **شدت درک شده** | | | | | |
| 1- سرطان دهانه رحم، بیماری خطرناکی است. |  |  |  |  |  |
| 2- سرطان دهانه رحم کشنده است. |  |  |  |  |  |
| 3- اگر کسی به سرطان دهانه رحم مبتلا شود، به رابطه زناشویی اش لطمه میخورد. |  |  |  |  |  |
| 4- حتی تصور ابتلا به سرطان دهانه رحم، مرا می ترساند. |  |  |  |  |  |
| 5- اگركسي سرطان دهانه رحم بگيرد، مدتها درگير مشكلات بيماري مي‌شود. |  |  |  |  |  |
| 6- ابتلا به سرطان دهانه رحم، زندگی فرد را مختل میکند. |  |  |  |  |  |

|  | **کاملاً مخالفم** | **مخالفم** | **نظری ندارم** | **موافقم** | **کاملاً موافقم** |
| --- | --- | --- | --- | --- | --- |
| **فواید درک شده برای ازمایش پاپ اسمیر** | | | | | |
| 1- آزمایش پاپ اسمیر، به تشخيص سرطان دهانه رحم در مراحل اولیه کمک می کند. |  |  |  |  |  |
| 2- آزمایش پاپ اسمیر، احتمال مرگ ناشي از سرطان دهانه رحم را كم مي‌كند. |  |  |  |  |  |
| 3- انجام آزمایش پاپ اسمیر باعث آسودگی خیال من می شود. |  |  |  |  |  |
| 4- آزمایش پاپ اسمیر، باعث شناسایی زودرس عفونت های رحم می شود. |  |  |  |  |  |

|  | **کاملاً مخالفم** | **مخالفم** | **نظری ندارم** | **موافقم** | **کاملاً موافقم** |
| --- | --- | --- | --- | --- | --- |
| **موانع درک شده برای ازمایش پاپ اسمیر** | | | | | |
| 1- از انجام آزمایش پاپ اسمیر خجالت مي كشم. |  |  |  |  |  |
| 2- وقت کافی جهت مراجعه برای آزمایش پاپ اسمیر ندارم. |  |  |  |  |  |
| 3- انجام آزمایش پاپ اسمیر برایم دردناک است. |  |  |  |  |  |
| 4- از انجام آزمایش پاپ اسمیر مي ترسم؛ چون نمي دانم چه کاری انجام میدهند. |  |  |  |  |  |
| 5- آزمایش پاپ اسمیر را انجام نمیدهم؛ چون می ترسم نتیجۀ آن مثبت باشد. |  |  |  |  |  |
| 6- در مورد کارایی پاپ اسمیر برای پیشگیری از سرطان دهانۀ رحم تردید دارم. |  |  |  |  |  |

|  | **کاملاً مخالفم** | **مخالفم** | **نظری ندارم** | **موافقم** | **کاملاً موافقم** |
| --- | --- | --- | --- | --- | --- |
| **خودکارآمدی درک شده برای ازمایش پاپ اسمیر** | | | | | |
| 1- من می توانم به راحتی اقدام به انجام آزمایش پاپ اسمیر بنمایم. |  |  |  |  |  |
| 2- من می توانم به راحتی برای انجام آزمایش پاپ اسمیر وقتم را تنظیم کنم. |  |  |  |  |  |
| 3- من می توانم به راحتی بر ترس خود از انجام آزمایش پاپ اسمیر غلبه کنم. |  |  |  |  |  |
| 4- من می توانم به راحتی بر خجالت خود از انجام آزمایش پاپ اسمیر غلبه کنم. |  |  |  |  |  |
